# Murine Tim-1 is excluded from the immunological synapse

**DOI:** 10.12688/f1000research.1-10.v2

**Published:** 2012-10-10

**Authors:** Jean Lin, Leo Chen, Lawrence P Kane

**Affiliations:** 1 University of Pittsburgh Medical Scientist Training Program and Graduate Program in Immunology, Pittsburgh, 15261, USA; 2 Department of Immunology, University of Pittsburgh School of Medicine, Pittsburgh, 15261, USA

## Abstract

The interaction between T cells and APCs bearing cognate antigen results in the formation of an immunological synapse (IS). During this process, many receptors and signaling proteins segregate to regions proximal to the synapse. This protein movement is thought to influence T cell function. However, some proteins are transported away from the IS, which is controlled in part by ERM family proteins. Tim-1 is a transmembrane protein with co-stimulatory functions that is found on many immune cells, including T cells. However, the expression pattern of Tim-1 on T cells upon activation by APCs has not been explored. Interestingly, in this study we demonstrate that the majority of Tim-1 on activated T cells is excluded from the IS. Tim-1 predominantly resides outside of the IS, and structure/function studies indicate that the cytoplasmic tail influences Tim-1 polarization. Specifically, a putative ERM binding motif (KRK 244-246) in the Tim-1 cytoplasmic tail appears necessary for proper Tim-1 localization. Furthermore, mutation of the KRK motif results in enhanced early tyrosine phosphorylation downstream of TCR/CD28 stimulation upon ectopic expression of Tim-1. Paradoxically however, the KRK motif is necessary for Tim-1 co-stimulation of NFAT/AP-1 activation and co-stimulation of cytokine production. This work reveals unexpected complexity underlying Tim-1 localization and suggests potentially novel mechanisms by which Tim-1 modulates T cell activity.

## Introduction

Antigen receptor, co-stimulatory, and signaling proteins adopt distinct patterns of localization and segregation upon T cell stimulation by peptide antigen presented by antigen-presenting cells (APC). Current models suggest that these patterns are critical for proper regulation of T cell activation. T cell recognition of an APC bearing cognate peptide drives the formation of a structure termed the immunological synapse (IS) or supramolecular activation cluster (SMAC) ^1^. In a "mature" synapse, many proteins important for transduction of TCR signaling concentrate at the center of the contact site, the central supramolecular activation cluster (cSMAC), between the T cell and APC. These proteins include CD3, CD28, ZAP-70 and PKC-θ ^1– 3^. At the IS, this concentration of signaling proteins may enhance signaling before engaged TCR’s are internalized, possibly to terminate signaling ^4^.

Opposite the immunological synapse is a region known as the distal pole complex (DPC). Many large adhesion and glycosylated molecules, such as CD43, are transported to this region ^4, 5^. Formation of the DPC is thought to be driven by ERM (ezrin, radixin, and moesin) family proteins ^6, 7^. The prevailing hypothesis is that the DPC serves as a reservoir for sequestering negative signaling molecules, such as CD43, away from the IS to allow for greater T cell activation ^8^. However, the presence of a pool of active signaling molecules, including ZAP-70, PIP _3_, and STIM-1 and Orai1 suggests an additional positive role for the DPC ^9– 11^. While the precise function of the DPC is disputed, formation of the DPC does appear to impact T cell activation. For example, disrupting localization of proteins to the DPC with an ERM dominant negative construct can disrupt specific functions, including transcriptional activation and cytokine production ^6, 12^.

Transmembrane immunoglobulin and mucin 1 (Tim-1) is a co-stimulatory molecule found on the surface of many immune cells. It was first identified in primates as a Hepatitis A virus cellular receptor (HAVCR1), although the mouse homolog does not bind HAV ^13^. Variants in murine (and human) Tim-1 were later associated with asthma susceptibility ^14– 16^. Early work on the immune function of Tim-1 also revealed a role for Tim-1 as a co-stimulatory molecule on CD4 ^+^ T helper cells by enhancing inducible transcription, cytokine production, and proliferation ^17, 18^. Tim-1 has also been implicated in the regulation of B cells, CD8 ^+^ T cells, dendritic cells, NKT cells, and mast cells ^19– 26^. Tim-1 antibodies have demonstrated efficacy in the modulation of immune function in different models of disease, including asthma and organ transplantation ^17, 19, 22, 27– 30^.

Although much is known about the effects of Tim-1 on transcription factor induction and cytokine production, less is known about the sub-cellular localization of Tim-1, especially in T cells. The function of many molecules has not been fully appreciated until their localization was understood. For example, the role of PKC-θ in T cells was greatly enhanced by the discovery that it localizes at the SMAC in effector T cells and away from the IS in regulatory T cells ^31, 32^. Understanding Tim-1 localization in T cells may provide similar insights into its function, particularly since some controversy still exists about the role of Tim-1 in T cell signaling. While previous studies have implicated Tim-1 in enhancing T cell activation ^17, 18, 33, 34^, one report has suggested that Tim-1 might function in either a positive or negative fashion, depending on the strength of antibody ligation ^35^. Another recent study demonstrated increased cytokine production by Tim-1 deficient T cells, suggesting that Tim-1 may also act as a negative regulator of T cell function, at least under some circumstances ^36^. Thus, defining Tim-1 localization on T cells under different conditions may yield novel insights that help to resolve these apparently disparate findings.

The localization of Tim-1 has not been extensively explored. A previous report suggested that Tim-1 exists in vesicles in the cytoplasm of human embryonic kidney cells (293) and 300.19 pre-B cells ^37^. Another study demonstrated that Tim-1 expressed on DO11.10 TCR transgenic T cells localized towards apoptotic thymocytes with exposed phosphatidylserine (PS), a Tim-1 ligand ^24^. Other studies have suggested that human TIM-1 interacts with ZAP-70 and PI3K ^33^ and may co-cap with CD3 ^35^.

At this point, relatively little is known about the sub-cellular localization of Tim-1, especially in T cells. In particular, where Tim-1 distributes (or re-distributes) upon T cell activation is poorly characterized. In this study, we define the patterns of Tim-1 localization before and after T cell recognition of antigen/MHC, as well as the functional consequences of altering Tim-1 localization. Our studies have revealed unexpected complexity in the regulation of Tim-1 localization and its function in T cell activation. These findings may have implications for understanding the function of Tim-1 in regulating immune responses.

## Materials and methods

### Reagents and cell culture

Jurkat, D10, Raji, and CH27 cell lines were used and cultured as previously described ^38^. The following antibodies were used: pSrc Y416 and pZAP-70 Y319 (Cell Signaling), PKC-θ (C-18, Santa Cruz), CD43 (clone S7, BD Pharmingen), M2-Cy3 (Sigma Aldrich), EEA1 (BD Transduction), M2 anti-flag (Sigma Aldrich), anti-human CD3 (Becton Dickinson), mouse CD3 and CD28 (BD Pharmingen), human CD28 (Life Technologies), Tim-1 Fc (eBiosciences), anti-TCR antibody C305 (Harlan), anti-Tim-1 antibodies (3B3 and 5F12), anti-Tim-4 antibodies (3A1, 3H11 and 5G3). Alexa fluor-conjugated secondary antibodies were from Life Technologies. Conalbumin was from Sigma Aldrich, and SEE from Toxin Technology.

### T cell: APC conjugates for confocal imaging

D10 cells were transiently transfected with up to 20 µg total of plasmid DNA by electroporation at 250V/950 µF and rested for 16 hours. Live cells were recovered the next day by spinning over a cushion of Lympholyte-M. D10 T cells (0.3×10 ^6^) were combined with an equal number of conalbumin-loaded CH27 cells by centrifugation at 3000 rpm for 3 minutes, followed by incubation at 37 degrees for 5–40 minutes. The pellet was gently resuspended by pipetting 3 times with a large-bore 1 ml pipet tip. Cells were allowed to settle on a poly-l-lysine coated coverslip for 20 min before being fixed at a final concentration of 2% PFA. Cells were permeablized with 0.1% TX-100 and were blocked for 30 min in 10% anti-goat or anti-donkey serum. The following were used: M2-Cy3 (2 µg/mL), pSyk/ZAP-70 (1:100), PKC-θ (1:100), and EEA1 (1:100). Secondary antibodies were used at the following concentrations: anti-rabbit Alexa-647 – 1:1000, anti-mouse Alexa-488 – 1:2000, anti-mouse Alexa-555 – 1:2000, anti-rat-Cy3 – 1:1000. Mid-plane images were captured on an Olympus FluoView 1000. Images were exported as bit TIFFs and analyzed with Image J. Figures were assembled in Canvas 8. For live cell imaging, Jurkat T cells were co-transfected with Tim-1 GFP and ZAP-70 Tag RFP. Equal numbers of Jurkat and SEE loaded Raji cells (0.075×10 ^6^ cells) were maintained at 37°C in Matek dishes and imaged on an Olympus FluoView 1000 or a Nikon A1.

Mature conjugates were identified by morphology and localization of ZAP-70 or PKC-θ at the interface between the B and T cell. Images were analyzed in Metamorph or Image J. When Tim-1 was localized opposite the IS, the cells were termed "anti-synapse". Tim-1 in conjugates that appeared close to the IS were termed "front half" of the cell. Tim-1 that appeared to be both opposite and near to the IS were termed "unpolarized". Finally, Tim-1 that had a predominantly intracellular and vesicular appearance was noted as "punctate".

For a select number of imaged conjugates that expressed Flag-Tim-1, two parameters were measured using Image J. First, the distance of Tim-1 from the synapse was determined as the angle between the center of the IS to the center of the Tim-1 region with the vertex of the angle set at the center of the cell. The extent of spread of Tim-1 was measured as the angle between the two edges of the Tim-1 region.

### Latex beads

8.7micron aldehyde/sulfate latex beads (Life Technologies) were prepared according to manufacturer instructions. 80×10 ^6^/mL beads were coated with 50 µg/mL anti-CD3 and 50 µg/mL anti-CD28.

### DNA constructs

Tim-1, Tim-1 Y276F, and Tim-1 cytoplasmic tail truncation were generated as described previously ^18^. Tim1-GFP was generated by inserting the C57Bl/6 Tim-1 into pEGFP-N1. Site-directed mutagenesis was utilized to mutate a Flag-Tim-1 construct, using the QuikChange system (Stratagene). The KRK at position 244–246 of the C57BL/6 allele of Tim-1 was mutated to QGQ using the following primers: Forward: cc aggta catac ttatg caagg gcagt cagca tctct aagcg; Reverse: cgctt agaga tgctg actgc ccttg cataa gtatg tacct gg. The sequence was verified by automated sequencing. The ERM DN-GFP construct was a gift from Dr. Janis Burkhardt. ZAP-70 cDNA and vector containing Tag RFP were gifts from Dr. Steven Bunnell.

### Flow cytometry

0.5×10 ^6^ Jurkat and D10 T cells were stained with 1 µg of M2 (anti-Flag) on ice and then stained with 1:200 Alexa-647 for surface staining. For intracellular staining cells were fixed in 1.5% paraformaldehyde at room temperature for 10 minutes. Cells were then permeablized on ice with ice cold methanol for 15 minutes before being washed and stained for M2 (anti-Flag). 0.5×10 ^6^ CH27 or Raji cells were stained with 1–4 ug of anti-Tim-1 or anti-Tim-4 antibodies on ice and then secondarily stained with 1:200 Alexa-647 on ice. Samples were read on a BD LSR II; FlowJo software was used to analyze data.

### Tyrosine phosphorylation western blotting

20×10 ^6^ Jurkat T cells were transfected with empty vector, Tim-1, or Tim-1 ^QGQ^. 1.5×10 ^6^ cells were lysed using 1% NP-40 lysis buffer in addition with beta-glycerophosphate, sodium fluoride, sodium orthovanadate, AEBSF, aprotinin, leupeptin, pepstatin (Calbiochem/EMD Biochemicals). Lysates were run on a 10% SDS-PAGE gel before being transferred to PVDF membrane and blotted with anti-pY (4G10). Blots were developed with Super-Signal Pico ECL (Pierce) and imaged on a Kodak Image Station 4000MM.

### TCR internalization

Jurkat cells were transfected as described above with pCDEF3 (empty vector), WT Tim-1, Tim-1 ^QGQ^, or Tim-1 lacking the cytoplasmic tail truncation (Tim-1 ^∆Cyto^). Cells were re-suspended at 0.5×10 ^6^ in PBS and placed on ice in the presence of anti-TCR (C305) at a dilution of 1:250 for 30 min. Cells were treated with 80 µM Dynasore for 20 minutes on ice to prevent clathrin-mediated TCR internalization. Cells were then incubated at 37°C for 0, 5, 10, 20, 30, 60, and 120 minutes. Immediately after the time points, cells were washed with ice cold PBS before staining with anti-human CD3 and M2 (to detect Flag Tim-1). Samples were read on a BD LSR II; data were analyzed using FlowJo software.

### Luciferase assays

Jurkat T cells were co-transfected with empty vector, WT Tim-1, or Tim-1 QGQ, along with an NFAT/AP-1 luciferase reporter construct. Cells were allowed to recover for 16 hours before stimulating with the anti-TCR antibody C305 (1:1000) in the presence or absence of anti-CD28 (1:100) for 6 hours at 37°. D10 T cells were co-transfected with empty vector, WT Tim-1, or Tim-1 QGQ, along with an NFAT/AP-1 luciferase reporter construct. Cells were allowed to recover for 16–18 hours before stimulating with 1 µg/mL biotinylated anti-CD3, -CD4 and -CD28, plus streptavidin for six hours at 37°. Luciferase activity was determined as described previously ^39^.

### ELISA

0.5×10 ^6^ Jurkat cells were stimulated with anti-TCR antibody C305 (1:1000), with or without CD28 (1:200) for 24 hours. Supernatants were taken and production of IL-2 was determined by ELISA (BD OptIA). D10 T cells (0.5×10 ^6^) were stimulated with 1 µg/mL anti-CD3, -CD4 and -CD28 for 24 hours before supernatants were collected for measurement of IL-4 and TNF-α by ELISA. Comparisons were analyzed by paired Student’s t test, performed with Prism.

### Limitations to data interpretation

The chief limitation in these studies is the subjective nature of data collection and interpretation in the confocal imaging experiments. To determine true positive signal, cells that were stained with comparable amounts of secondary antibodies alone were compared to staining with primary and then secondary antibodies. In addition, the laser voltage was adjusted so that pixels were not saturated. Protein localization was observed in two different T cell lines, using both epitope- and GFP-tagged constructs. The core observation of WT mTim-1 exclusion from the immune synapse was observed over the course of dozens of experiments. Quantitation of WT and mutant Tim-1 localization was pooled from conjugates obtained in multiple separate experiments. Other experiments were performed at least three times, with statistical analysis being performed on replicates within a representative experiment.

## Results

### Tim-1 is excluded from the immunological synapse

**Figure 1.**
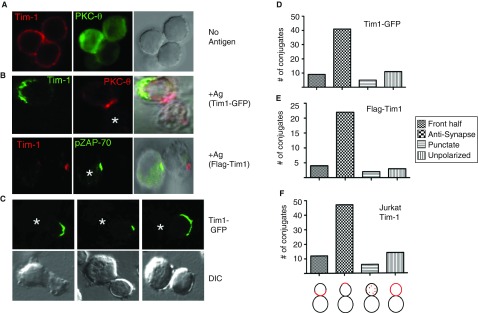
Tim-1 re-distributes away from the immunological synapse. ( **A**) Resting D10 T cells transiently co-transfected with Flag-Tim-1 and PKC-θ-GFP (green) were fixed, stained with anti-Flag-Cy3 (red), and visualized mid-plane by confocal microscopy. ( **B** - upper panels) D10 T cells transiently transfected with Tim-1-GFP (green) were conjugated with conalbumin-loaded CH27 B cells. Endogenous PKC-θ was stained with PKC-θ(C-18) and Alexa-555-conjugated secondary antibody (red) as a marker of the IS/c-SMAC. ( **B** - lower panels) D10 T cells transiently transfected with Flag-Tim1 (red) were conjugated with antigen loaded CH27 cells and stained with pZAP-70 and Alexa 488-conjugated secondary antibody (green) as a marker of the cSMAC. ( **C**) Additional D10 T cell:APC conjugates showing the exclusion of Tim1-GFP from the IS. ( **D**) Quantitation of the phenotype of Tim-1 GFP localization in D10:CH27 conjugates (n=66) from 15 experiments or ( **E**) Flag-Tim-1 in D10:CH27 conjugates (n=31) from 6 experiments. ( **F**) Quantitation of Tim-1 localization on Jurkat T cells making synapses with superantigen-loaded Raji B cells. ( **F**, bottom) Schematic of system used to score conjugate phenotypes.

To define patterns of Tim-1 localization on T cells, we transfected Tim-1 into the murine Th2 line D10, which does not expresses endogenous Tim-1 ^18^. In contrast to studies that reported predominantly intracellular Tim-1 in non-T cells ^37^, we found Tim-1 diffusely expressed on the surface of resting T cells ( Figure 1A). However, when Tim-1 expressing T cells are activated by antigen loaded APCs; the pattern of Tim-1 localization is altered. Surprisingly, Tim-1 concentrates in a region opposite the immunological synapse, with the latter represented by PKC-θ or pZAP-70 (Y319). This localization is not epitope tag-dependent, since both C-terminus tagged Tim1-GFP and N-terminus tagged Flag-Tim1 localize opposite, or at least outside, the immunological synapse ( Figure 1B). The majority of Tim-1 in T cell:APC conjugates (51.25% and 71% with Tim1-GFP and Flag-Tim1, respectively) appears in the "back" half of the cell, opposite, or at least away from, the immunological synapse ("anti-synapse"; Figure 1C–E). Tim-1 was present within the IS in only 1 conjugate (1.03% of the total). This appears to be a general phenomenon, as Tim-1 localization on Jurkat T cells interacting with APC’s is also found predominantly outside of the immunological synapse (57% of conjugates; Figure 1F).

**Figure 2.**
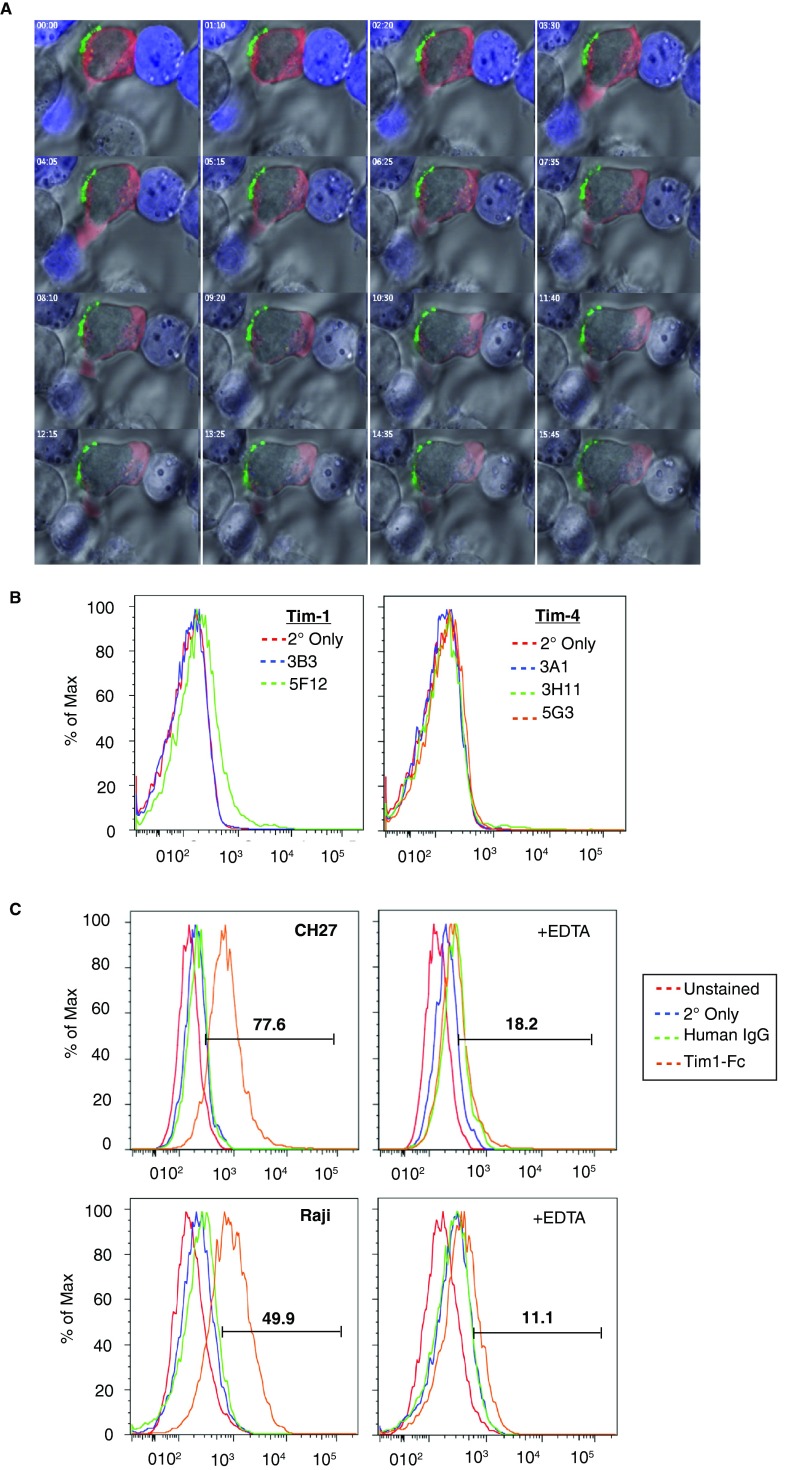
Tim-1 is excluded from the IS despite Tim-1 ligand(s) on the APC. ( **A**) Jurkat T cells transiently transfected with Tim-1-GFP (green) and ZAP70-Tag-RFP (red) were incubated with Raji cells pre-loaded with 1 µg/mL SEE and stained with Cell Tracker Blue (blue). Cells were incubated in a heated chamber for live cell imaging. ( **B**) CH27 cells were stained with anti-Tim-1 (left) and anti-Tim-4 (right) antibodies and secondary antibody and analyzed by flow cytometry. ( **C**) The presence of Tim-1 ligand(s) on CH27 (upper panels) and Raji (lower panels) B cells was revealed by staining with Tim1-Fc and secondary antibody, in the absence (left) or presence (right) of EDTA, followed by flow cytometry.

To further demonstrate that Tim-1 localizes predominantly away from the cSMAC, we performed live cell microscopy. Again, Tim-1 moved away from the nascent ZAP70-containing immunological synapse ( Figure 2A and Movie 1).

WT Tim-1 moves away from the nascent IS after APC stimulation Jurkat T cells transiently transfected with Tim-1-GFP (green) and ZAP70-TagRFP (red) were incubated with Raji cells incubated with 1 mg/mL SEE and stained with Cell Tracker Blue (blue). Mid-plane images were taken every 6.7 seconds over the course of 19 minutes in a heated chamber on an Olympus FluoView 1000 confocal microscope.

We also utilized a more reductionist system to examine the effect of anti-TCR and –CD28 on Tim-1 localization. Thus, Jurkat T cells expressing Tim1-GFP and ZAP70-TagRFP were mixed with latex beads coated with anti-CD3/CD28 antibodies. Here we observed that Tim-1 initially appears to concentrate near the bead along with ZAP-70. However, over time, most Tim-1 moves away from the beads ( Movie 2). Overall, the pattern of Tim-1 localization is reminiscent of the distal pole complex ^8^.

Imaging Tim-1 movement in response to latex beads Jurkat T cells transiently co-transfected with Tim-1-GFP (green) and ZAP70-TagRFP (red) were incubated with anti-OKT3, anti-CD28 coated latex beads. Mid-plane images were taken every 23 seconds over the course of 19 minutes in a heated chamber on an Olympus FluoView 1000 confocal microscope.

Some proteins require the expression of their ligands on the APC in order to localize towards the IS. For instance, CD28 only localizes to the cSMAC in the presence of APCs expressing of one of its ligands – CD80 or CD86 ^40^. In agreement with the importance of ligands in receptor localization, it has been shown that Tim-1 faces apoptotic cells bearing one of its ligands, i.e. phosphatidylserine ^24^. We tested whether the APCs used in our system contain a ligand for Tim-1. Variable results were obtained when probing for expression of known ligands for Tim-1, including Tim-1 itself and Tim-4, on the APCs that we employed, CH27 and Raji ( Figure 2B and data not shown). While Tim-4 was consistently *not* detected on CH27 cells, Tim-1 staining was more variable. We did confirm that both types of APCs used in our studies express one or more surface ligands for Tim-1, as evidenced by binding of Tim1-Fc to the surface of the APCs ( Figure 2C). Furthermore, Tim1-Fc binding to these cells was abolished in the presence of EDTA, demonstrating that Tim1-Fc binding to this/these still-undefined ligand(s), like the known Tim-1 ligands, requires divalent ions ^41, 42^. This finding is not entirely surprising since unidentified Tim-1 ligands have been suggested to exist in a previous study ^43^. Thus, although known Tim-1 ligands (Tim-1/Tim-4) may or may not be expressed on the surface of the APCs used in our studies, one or more Tim-1 ligand(s) are present. Interestingly, this still does not result in Tim-1 localization towards the IS.

### Structural requirements for proper Tim-1 localization

Next, we determined the elements necessary for Tim-1 localization away from the IS. During conjugate formation, many proteins depend on motifs found in the cytoplasmic tail for proper localization. For instance, CD28 requires Y188 in its cytoplasmic tail for localization towards the IS ^44^. Likewise, CD43, which moves opposite the immunological synapse and to the distal pole complex, requires its cytoplasmic tail for this localization ^6^. Specifically, CD43 requires a membrane-proximal positively charged amino acid cluster (KRR) in its cytoplasmic tail for ERM binding and distal pole complex localization ^45^. ERM family proteins are necessary for driving certain proteins, such as CD43 and Rho-GDI, towards the DPC ^6, 46^. Intriguingly, we noted a similar sequence in the juxtamembrane region of the Tim-1 cytoplasmic tail – a KRK motif at residues 244–246.

**Figure 3.**
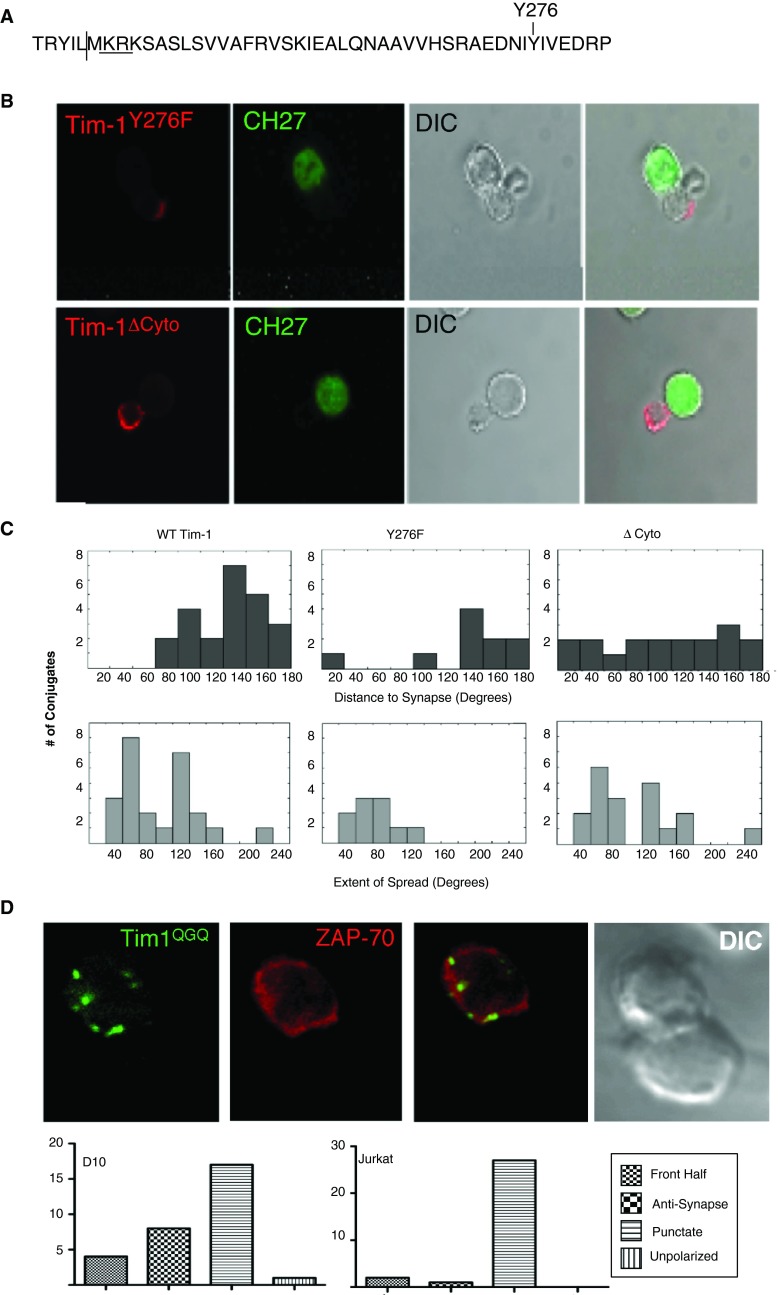
The cytoplasmic tail regulates Tim-1 localization relative to the IS. ( **A**) Murine Tim-1 cytoplasmic tail sequence. The vertical line indicates the location of the truncation in the delta-cyto construct. KRK is the putative ERM binding domain; Y276 is underlined. ( **B**) D10 T cells transiently transfected with either Flag-Tim1 ^Y276F^ (red) or Flag-Tim-1 cytoplasmic tail truncation (del.cyto; red) were mixed with conalbumin loaded CH27 cells (green). Cells were then stained with anti-Flag mAb directly conjugated to Cy3. ( **C**) Quantitation of the angle from the IS to Tim-1 (top) and the extent of distribution of Tim-1 on the cell surface (bottom). ( **D** - upper) Representative image of Tim-1 ^QGQ^-GFP and ZAP-70 RFP expressing D10 cells interacting with antigen-loaded CH27 B cells. ( **D** - lower) Quantification of Tim-1 ^QGQ^ localization in D10:CH27 and Jurkat:Raji conjugates from 12 and 13 experiments, respectively.

To determine the intrinsic requirements for Tim-1 exclusion from the IS, we examined the effect of three constructs on Tim-1 localization. Specifically, we tested the effect of Tim-1 ^Y276F^, a cytoplasmic tail truncation (Tim-1 ^del.cyto^), and Tim-1 244–246 KRK-QGQ (Tim-1 ^QGQ^) on Tim-1 localization ( Figure 3A). As shown previously by our group, Y276 is critical for Tim-1 co-stimulatory function ^18^. However, the Tim-1 ^Y276F^ mutant construct still appears to localize opposite the immunological synapse ( Figure 3B). To quantify the location and extent of spread of Tim-1 we examined two parameters. First, to determine the distance of Tim-1 in relation to the IS of T cell:APC conjugates, we measured the angle of Tim-1 from the center of the IS. Thus, if Tim-1 were concentrated directly opposite the synapse, Tim-1 would be 180° away from the IS. Second, we measured the extent of Tim-1 spreading on the cell surface. Wild type Tim-1 is predominantly found in the "back" half of the cell (>90 degrees away from the synapse with a median of 133.3°), opposite the immunological synapse, and is fairly tightly contained (spread of 20–180° with a median of 79.6°) ( Figure 3B–C). Tim-1 ^Y276F^ localization is similar to wild type Tim-1, in that in a majority of conjugates the protein is found more than 90° (median 136.6°) from the synapse and is spread over 20–120° with a median of 58.1° ( Figure 3B–C). These findings suggest that the majority of Tim-1 ^Y276F^ is concentrated opposite the synapse. Next, a Tim-1 cytoplasmic tail truncation was utilized. In contrast to WT or Y276F forms of the protein, Tim-1 with a cytoplasmic tail truncation is more likely to be present in the front half of the cell, closer to the IS with a median distance from the IS of 106.5° ( Figure 3C). In about half of the conjugates analyzed, the Tim-1 ^del.cyto^ construct was found in the front half of the cell (less than 90° from the IS), and in 28% of total conjugates Tim-1 ^del.cyto^ even appears to cross into the IS ( Figure 3C).

The greatest change in Tim-1 localization that we have observed thus far is seen when the positively charged, putative ERM-binding, motif in Tim-1 (244–246 KRK) is mutated. Rather than localizing diffusely on the surface of the T cells, Tim-1 ^QGQ^ has a predominantly punctate (56.7% of D10 conjugates and 90% of Jurkat conjugates) appearance, consisting of mainly intracellular Tim-1, with some of this mutant even present in the IS ( Figure 3 and Movie 3). Thus, the ability of Tim-1 to bind ERM proteins appears to be important for Tim-1 localization distal to the IS and within the DPC.

Tim-1QGQ localizes to intracellular pools that can reside at the IS Jurkat T cells transiently transfected with Tim-1QGQ-GFP (green) and ZAP70-TagRFP (red) were incubated with Raji cells incubated with 1mg/mL SEE and stained with Cell Tracker Blue (blue). Mid-plane images were taken every 6.7 seconds over the course of 14 minutes in a heated chamber on an Olympus FluoView 1000 confocal microscope.

### Tim-1 co-localizes with ERM proteins

**Figure 4.**
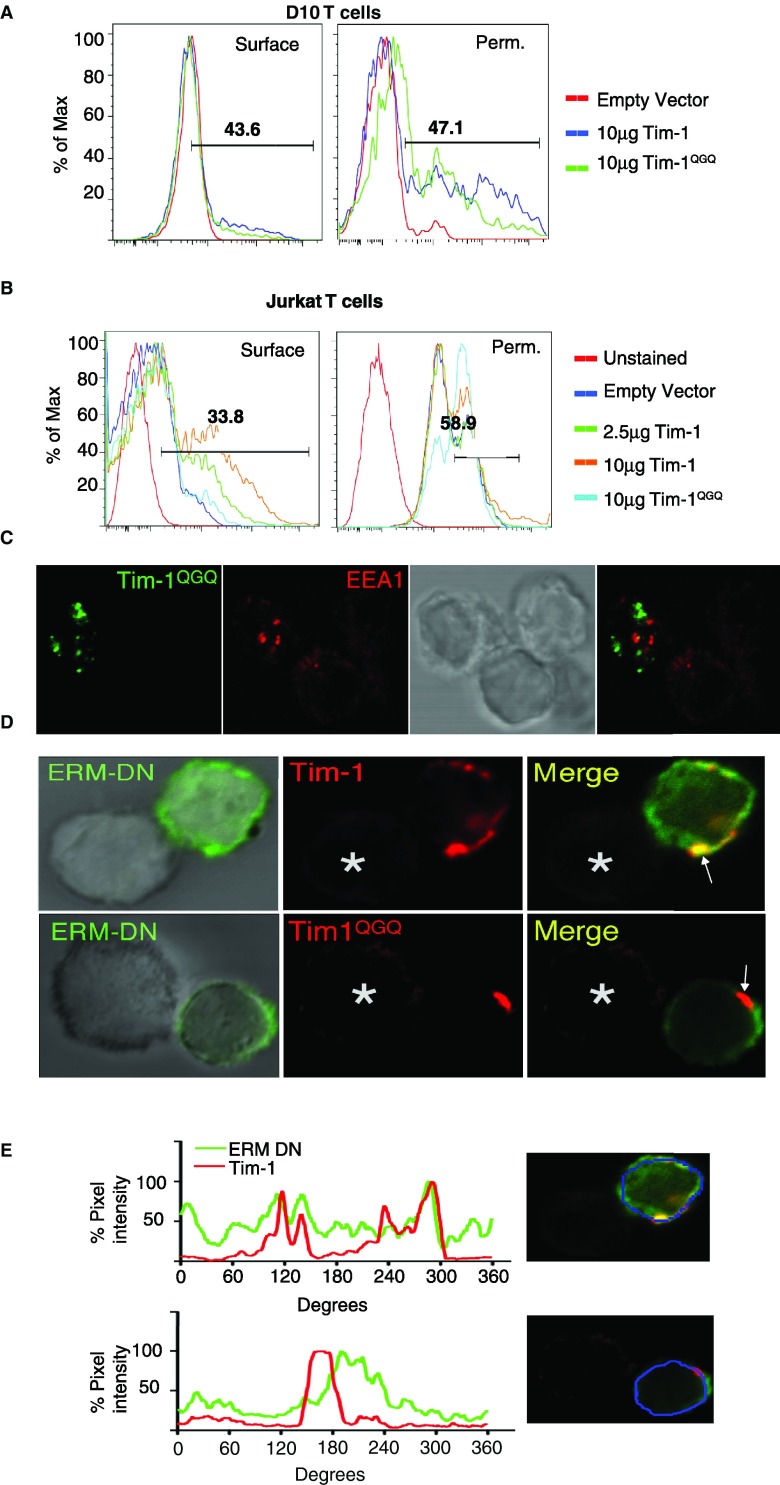
A putative ERM-binding motif in the cytoplasmic tail regulates Tim-1 localization. Anti-Flag staining of EV (empty vector), Flag-Tim-1, or Flag-Tim-1 ^QGQ^ transfected D10 ( **A**) or Jurkat T cells ( **B**) as determined by flow cytometry. Surface staining of non-permeablized cells is on the left. Methanol permeabilization of T cells for intracellular Flag expression of EV (empty vector), Flag-Tim-1, or Flag-Tim-1 ^QGQ^ transfected T cells, as determined by flow cytometry (right). ( **C**) Representative image of Jurkat T cells transiently transfected with Tim-1 ^QGQ^-GFP (green) and co-stained for EEA1 and Alexa-555 (red) after conjugation to antigen loaded Raji cells from three experiments. ( **D**) D10 T cells co-transfected with Flag-Tim1 or Flag-Tim1 ^QGQ^ (red) and FERM-GFP ("ERM-DN") constructs and conjugated to antigen-loaded CH27 cells were stained with anti-Flag-Cy3 antibody and imaged by confocal microscopy. ( **E**) To quantify the ERM DN and Tim-1 localization, a ten pixel line scan was drawn along the surface of the cell, with the intensity of staining represented as a percentage of the maximal pixel intensity. Top – WT Tim-1; Bottom – Tim-1 ^QGQ^.

Given the dramatic effect on Tim-1 localization, we further characterized the Tim-1 ^QGQ^ mutant. We observed that the Tim-1 ^QGQ^ construct has lower surface expression than wild type Tim-1, even when higher concentrations of Tim-1 ^QGQ^ plasmid are transfected. Although the total amount of plasmid transfected is the same (10 µg total), 10 µg of Tim-1 ^QGQ^ plasmid yields less surface expression than 2.5 µg of WT Tim-1 plasmid (along with 7.5 µg empty vector). However, the total amount of Tim-1 ^QGQ^ protein appears to be equivalent to WT when cells are permeablized ( Figure 4A–B). This is consistent with our imaging, wherein Tim-1 ^QGQ^ is not highly expressed on the cell surface but appears to distribute into intracellular pools within the cell ( Figure 3D). Further, Tim-1 ^QGQ^ does not co-localize with early endosomal antigen 1 (EEA1), suggesting that this pool of vesicular Tim-1 is not found in early endosomes ( Figure 4C). The significant amount of intracellular Tim-1 ^QGQ^ suggests either that Tim-1 ^QGQ^ is rapidly recycled from the cell surface or that Tim-1 ^QGQ^ is retained in a vesicular compartment within the cell.

Since the KRK sequence in the Tim-1 cytoplasmic tail represents a putative ERM binding motif, we wanted to determine whether Tim-1 might interact with ERM proteins. Here, we used an dominant negative (DN) ERM construct, containing the N-terminal FERM domain (from ezrin) that binds proteins with ERM-binding motifs, along with a GFP moiety, but not the C-terminal actin-binding domain ^6^. When cells are co-transfected with both Tim-1 and the ERM-DN, there is partial Tim-1 co-localization with the FERM-GFP ( Figure 4D–E). This is consistent with a role for WT Tim-1 interacting with ERM proteins in the regulation of Tim-1 localization. However, mutation of the Tim-1 KRK motif diminishes the ability of the mutant to interact with the FERM-GFP construct, as compared to WT Tim-1 ( Figure 4D–E), providing further validation of a possible interaction between Tim1- and ERM family proteins.

### Altering Tim-1 localization impacts its effects on early tyrosine phosphorylation

**Figure 5.**
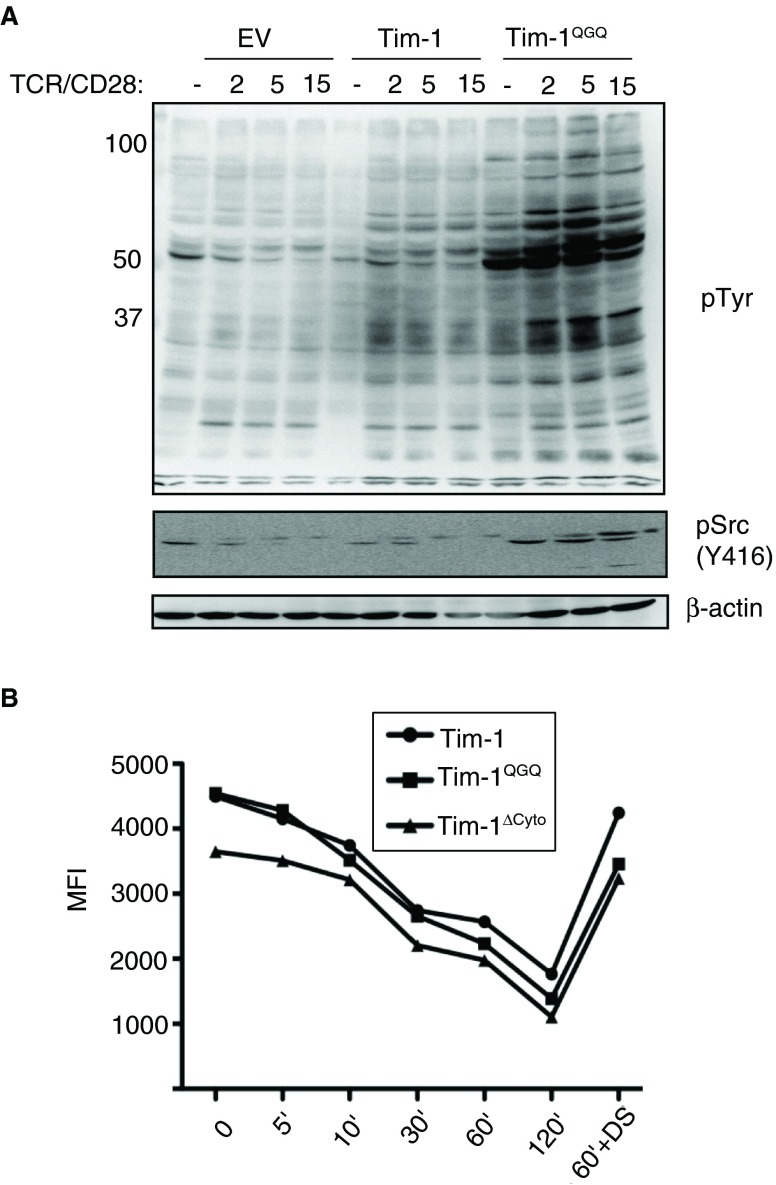
Tim-1 ^QGQ^ expression enhances early signaling events downstream of TCR/CD28 independent of the rate of TCR internalization. ( **A**) Jurkat T cells transfected with empty vector (EV), Flag-Tim-1, or Flag-Tim-1 ^QGQ^ were stimulated with anti-TCR and anti-CD28 antibodies for the indicated times. Lysates were analyzed by SDS-PAGE and western blotting for pY (4G10), pSrc (Y416; analogous to Y394 in Lck), and β-actin. ( **B**) Jurkat T cells were stimulated with anti-CD3 mAb for the indicated times. CD3 expression was measured with flow cytometry and mean fluorescence intensity (MFI) was determined in FloJo. Dynasore (DS, 80 µM) was used to prevent clathrin-mediated endocytosis after TCR/CD3 crosslinking.

We next determined whether Tim-1 localization affects Tim-1 co-stimulatory activity in conjunction with TCR and CD28. Interestingly, we were surprised to find that expression of Tim-1 ^QGQ^ promotes enhanced cellular tyrosine phosphorylation, as compared to wild type Tim-1 ( Figure 5A). One of the tyrosine phosphorylated substrates induced in the Tim-1 ^QGQ^ expressing cells is a band slightly above 50 kD. Since this would be consistent with Src family kinases (SFK), we were interested in determining whether this band was a phosphorylated SFK member. Using antibodies against the activating tyrosine (Y416 in Src), we were able to detect increased phosphorylation in Tim1 ^QGQ^ - expressing cells within minutes of TCR/CD28 stimulation ( Figure 5A). Although not all tyrosine phosphorylation results in positive signaling, the increased phosphorylation at the activating tyrosine (Y416 in Src) in T cells expressing the Tim-1 ^QGQ^-expressing cells suggests that Tim-1 ^QGQ^ may enhance early T cell signaling.

Since two previous reports demonstrated an association between Tim-1 and CD3 ^33, 35^, another possible explanation for the enhanced tyrosine phosphorylation in Tim-1 ^QGQ^ expressing cells was that Tim-1 ^QGQ^ might increase the levels of surface TCR/CD3 and/or slow the rate of TCR/CD3 internalization. To address this possibility, we stimulated Jurkat cells expressing WT or mutant Tim-1 with anti-CD3 antibody, and measured the levels of CD3 surface expression by flow cytometry. As expected, after antibody crosslinking, CD3 surface expression decreased over time ( Figure 5B). T cells expressing WT Tim-1 or Tim-1 ^QGQ^ displayed equivalent rates of TCR internalization, although starting levels of TCR/CD3 varied somewhat ( Figure 5B). Thus, impairment of TCR/CD3 down-regulation does not appear to be the mechanism behind the increased tyrosine phosphorylation in T cells expressing Tim-1 ^QGQ^ after CD3 crosslinking.

### Tim-1 ^QGQ^ is impaired in co-stimulation of inducible transcription and cytokine production

**Figure 6.**
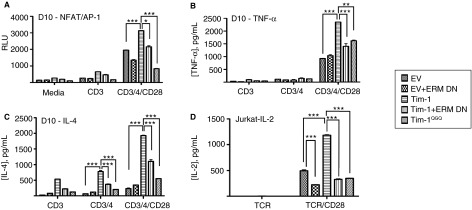
Altered Tim-1 localization impacts inducible transcription and cytokine production. ( **A**) D10 cells were transfected with an NFAT/AP-1 reporter, along with empty vector, WT or mutant Flag-Tim-1 in the presence or absence of ERM-DN. The next day, cells were cultured for six hours in the presence or absence of CD3/CD4/CD28 stimulation before assaying for luciferase activity. ( **B**) D10 T cells were transfected with empty vector, WT Tim-1, ERM-DN, or Tim-1 ^QGQ^. Cells were stimulated with anti-CD3 or anti-CD3/CD28 antibodies for 24 hours. Cell-free supernatants were collected and assayed for TNF-α production by ELISA. ( **C**) D10 T cells were transfected with empty vector, Tim-1, ERMDN, or Tim-1 ^QGQ^. Cells were stimulated with anti-CD3 or anti-CD3/CD28 antibodies for 24 hours and IL-4 production was determined by ELISA. ( **D**) Jurkat T cells were transfected with empty vector, Tim-1, ERMDN, or Tim-1 ^QGQ^. Cells were stimulated with α-TCR or α-TCR/CD28 for 24 hours before IL-2 production was determined by ELISA. Data are presented as average values, +/- standard deviation, of duplicate samples from an individual experiment.

Next, we examined the effects of altering Tim-1 localization on TCR-induced transcription and cytokine production. As we demonstrated previously, ectopic expression of WT Tim-1 leads to increased TCR-induced activation of an NFAT/AP-1 transcriptional reporter ^18, 34^. However, Tim-1 ^QGQ^ was not able to enhance NFAT/AP-1 activation ( Figure 6A). Furthermore, while expression of WT Tim-1 is associated with enhanced cytokine production, expression of Tim-1 ^QGQ^ is not ( Figure 6B–D). Consistent with a role for ERM protein binding to the KRK motif in Tim-1, a dominant negative ERM construct also suppresses the ability of WT Tim-1 expression to enhance transcription or cytokine production ( Figure 6). These findings suggest that Tim-1 interaction with ERM proteins, with proper subsequent localization of Tim-1, plays a role in Tim1-mediated transcriptional activity and cytokine production.

## Discussion

Here, we have shown that in contrast to the majority of known co-stimulatory molecules and TCR associated signaling molecules, Tim-1 does not localize towards the immunological synapse. Rather, surprisingly, Tim-1 is excluded from the immunological synapse in an ERM-dependent manner. Our structure/function studies suggest that Tim-1 exclusion from the immunological synapse is an active process requiring more than one step. First, the Tim-1 cytoplasmic tail appears to be necessary for exclusion from the immunological synapse, since a cytoplasmic tail truncation results in greater amounts of Tim-1 in the SMAC. Second, specific residues in the cytoplasmic tail (i.e. KRK) are required for proper Tim-1 localization towards the distal pole complex. Furthermore, concentration of Tim-1 opposite the immunological synapse towards the anti-synapse, or distal pole complex, appears to influence both early signaling and Tim-1 induced enhancement of T cell function. While most of our studies have employed a murine T cell clone (D10), it remains to be seen whether such patterns of Tim-1 localization hold true for all situations in which Tim-1 is expressed by T cells.

We find that Tim-1 is found mostly on the cell surface of T cells in the steady state. This is in contrast to previously published reports suggesting that Tim-1 is maintained in a mostly intracellular store and only becomes localized to the cell surface upon activation ^37^. These discrepancies could be due to differences in cell type. Thus, the previously published report used HEK 293 cells and 300.19 pre-B cells. Also, since Tim-1 is a transmembrane protein, it is also possible that WT Tim-1 might reside in an intracellular compartment before being inducibly cycled to the surface, similar to CLTA-4. On T cells, Tim-1 localizes towards the interface with PS-expressing apoptotic thymocytes, a finding we were also able to confirm (data not shown) ^24^. However, in our studies Tim-1 does not localize towards the interface with APCs bearing antigenic peptide and an unidentified Tim-1 ligand. This suggests that different Tim-1 ligands may have distinct effects on localization. Further examination of known Tim-1 ligands, such as Tim-4 and HAV, may help to clarify this issue. In addition, it will also be of interest to determine the identity of the as-yet-unknown ligand(s) expressed on the B cell lines that we have used as APC’s in our studies.

Regarding the relationship of Tim-1 to TCR/CD3, there is some discrepancy between our findings and the recent literature. Thus, it has been suggested that hTIM-1 co-localizes with CD3 and ZAP-70 and that CD3 can be co-capped with mTim-1 ^33, 35^. These findings suggest that Tim-1 should be found at the IS with CD3 and ZAP-70. However, none of the previous studies investigated the kinetics of Tim-1 localization or its localization on T cells in conjugates with antigen-bearing APCs. We have shown that Tim-1 may at least partially co-localize with ZAP-70 in the presence of TCR/CD28 coated beads (and absence of any ligand for Tim-1). However, at later time points Tim-1 relocates away from the antibody coated beads. In addition, we have obtained preliminary data indicating that Tim-1 and ZAP-70 microclusters may co-localize (data not shown). This suggests that Tim-1 and ZAP-70 might interact at some early time point during T cell activation but that the interaction may not persist.

The question then arises of the functional importance of Tim-1 exclusion from the IS, possibly at the distal pole complex in T cells, and how it might relate to Tim-1 enhancement of NFAT/AP-1 activation and cytokine production. If Tim-1 is truly a co-stimulatory molecule, then why would it be excluded from the IS? While the predominant view in the field is that the region opposite the IS, or distal pole complex, serves as a reservoir for molecules that inhibit signaling, there is evidence that the DPC may also serve as an area for active signaling. Multiple reports in the literature have shown that certain active signaling molecules, including PIP _3_, ZAP-70, STIM1/Orai, and CD46, reside at least in part in the DPC ^9– 11, 47^. Thus, Tim-1 may localize in the DPC in order to avoid being internalized and degraded at the immunological synapse. This may allow for extended time to interact with other signaling molecules, and in this way enhance signaling. Alternatively, Tim-1 may also enhance signaling by binding inhibitory molecules and moving them towards the DPC and away from the positively acting signaling molecules found at the immune synapse. However, it should be noted that one of the recentTim-1 knockout studies suggested that Tim-1 defcient mice develop worse lung inflammation in a model of airway hyper-reactivity, although another knockout study did not demonstrate this ^36, 48^.

Also intriguing is the paradoxical difference between early signaling events in cells expressing WT Tim-1 or Tim-1 ^QGQ^. Surprisingly, Tim-1 ^QGQ^-expressing cells displayed enhanced tyrosine phosphorylation at early time points downstream of TCR and CD28 stimulation, compared with the effects of WT Tim-1. This may represent phosphorylation of inhibitory molecules and/or increased tyrosine phosphorylation of positive signaling molecules. Also, the punctate appearance of Tim-1 ^QGQ^ could result from localization in endosomal compartments. Recent studies have highlighted the importance of endosomal vesicles carrying signaling molecules (e.g. LAT) into microclusters, in order to enhance the very earliest signaling events at the microclusters ^49^. Thus, vesicular Tim-1 ^QGQ^ localization could enhance early signaling before being rapidly transmitted to the SMAC for degradation. In this way, Tim-1 ^QGQ^ may enhance very early signaling and be degraded before having an opportunity to enhance later events, such as cytokine production and transcriptional activity. Alternatively, the Tim-1 ^QGQ^ mutant may be rapidly internalized, which would explain the reduced levels of surface expression. Proximal to the Tim-1 KRK motif is a YILM motif that is very similar to the CTLA-4 clathrin adaptor-binding motif (YVKM) ^50^. It is therefore possible that the KRK-QGQ mutation (and subsequent reduced ERM protein binding) exposes this YILM motif and causes increased internalization. This would also be consistent with the fact that the Tim-1 ^del.cyto^ construct, in which part of this motif is truncated (before the M), is not found in an intracellular, vesicular, compartment. WT Tim-1 may also briefly cycle through these endosomal compartments before being expressed more stably at the cell surface. Since a recent report has suggested that internalized/endosomal TCR can signal, it is possible that the increased early tyrosine phosphorylation in cells expressing Tim-1 ^QGQ^ arises from this internal compartment ^51^.

Relevant for this discussion, recent reports have also implicated signaling from endosomes as contributing to lymphocyte activation ^49, 51, 52^. Tim-1 ^QGQ^ displays a predominantly punctate pattern, which is consistent with possible endosomal localization. Thus, another intriguing possibility is that during early signaling events, Tim-1 ^QGQ^ in endosomes can enhance early signaling events downstream of the TCR. Thus, future studies will investigate the effects of WT and QGQ forms of Tim-1 on the localization of proximal TCR signaling proteins.

The movement of proteins during T cell interaction with antigen presenting cells impacts T cell function. Here we have demonstrated that Tim-1 on T cells preferentially localizes opposite the immunological synapse when conjugated to antigen-bearing APCs. Our studies have begun to unravel the motifs and complexities involved with regulating Tim-1 localization. These findings may provide insight into the mechanism underlying the effects of Tim-1 on the immune response.
