# Supplementary material for: Murine Tim-1 is excluded from the immunological synapse
Source: F1000Res. 2012 Oct 10;1:10. Originally published 2012 Aug 10. [Version 2] doi: 10.12688/f1000research.1-10.v2 (PMC3938182; doi:10.12688/f1000research.1-10.v2)
Supplement: Peer review history

# Peer review: Referee response for version 2

**Published:** 2012-10-19

**Authors:** Wenxia Song

**Affiliations:** 1 Department of Cell Biology and Molecular Genetics, University of Maryland, MD, 20742, USA

I am now satisfied with the changes that have been made by the authors, and these revisions have made an improvement from the first version of the article.

There are a few other minor concerns that the author may want to be consider;

1. All the data presented in this manuscript are generated by T cell lines or T cell “clone”. D10 cells are T lymphoblasts that continuously proliferate without stimulation, thereby exhibiting cancer cell properties. It is important to demonstrate this phenomenon occuring in vivo using primary T cells.

2. It isn’t very unclear how TCR internalization was measured by using anti-TCR antibody in the beginning and the end of incubation; this could be explained better.

3. In the abstract, it would read better if the word “interestingly” was deleted from the start of the second paragraph.

I have read this submission. I believe that I have an appropriate level of expertise to confirm that it is of an acceptable scientific standard.

# Peer review: Referee response for version 1

**Published:** 2012-09-06

**Authors:** Terry Strom

**Affiliations:** 1 The Transplant Institute, Beth Israel Deaconess Medical Center, Boston, MA 02115, USA

The findings concerning the role of TIM1 and domains thereof in the trafficking of T cells are original and important.

The manuscript is clear and uses powerful tools. I do have minor reservations on the discussion concerning apparent differences between findings in this paper and those reported elsewhere. The authors use a simple reductionist tool kit. Normal T cells are not studied (for reasons that are well justified). Differences in the cell population used in this and the cited works may or may not prove important. Next is the choice reagents to conduct the analysis. In mouse systems there are anti Tim1 antibodies that stimulate and others that inhibit T cell activation. It would be fascinating to learn (not in this paper) whether this is due to effects on Tim1 localization in or opposed to the IS.

I have read this submission. I believe that I have an appropriate level of expertise to confirm that it is of an acceptable scientific standard.

# Peer review: Referee response for version 1

**Published:** 2012-08-29

**Authors:** Wenxia Song

**Affiliations:** 1 Department of Cell Biology and Molecular Genetics, University of Maryland, MD, 20742, USA

The research described in this article is interesting, mostly well done, and worth publishing.

My major concerns:1. The manuscript over interprets some of the data.2. The polarized distribution of Tim1 needs a confirmation using primary T cells.3. The effects of Tim1 mutations on the distribution of signaling molecules should be included to support tyrosine phosphorylation data.

I have read this submission. I believe that I have an appropriate level of expertise to confirm that it is of an acceptable scientific standard, however I have significant reservations, as outlined above.

# Response

**Published:** 2012-08-30

**Authors:** Lawrence Kane

**Affiliations:** Department of Immunology, University of Pittsburgh School of Medicine, USA

Thanks for the feedback on our ms. Could you please clarify what you mean in point #1, regarding which data you think we should interpret more conservatively?
